# Women Veterans’ Experience With a Web-Based Diabetes Prevention Program: A Qualitative Study to Inform Future Practice

**DOI:** 10.2196/jmir.4332

**Published:** 2015-05-25

**Authors:** Tannaz Moin, Kristyn Ertl, Jessica Schneider, Elena Vasti, Fatima Makki, Caroline Richardson, Kathryn Havens, Laura Damschroder

**Affiliations:** ^1^VA Health Services Research and Development (HSR&D) Center for Healthcare Innovation, Implementation, and Policy, VA Greater Los AngelesVA Greater Los Angeles Healthcare System, Los Angeles, CADavid Geffen School of Medicine, University of California, Los Angeles, CALos Angeles, CAUnited States; ^2^Department of Research, Clement J. Zablocki VA Medical Center, Milwaukee, WICenter for Patient Care and Outcomes Research, Medical College of Wisconsin, Milwaukee, WIMilwaukee, WIUnited States; ^3^Department of Medicine, Medical College of Wisconsin, Milwaukee, WIWomen’s Health Clinic, Clement J. Zablocki VA Medical Center, Milwaukee, WIMilwaukee, WIUnited States; ^4^VA Greater Los Angeles Healthcare SystemLos Angeles, CAUnited States; ^5^VA Ann Arbor Center for Clinical Management ResearchAnn Arbor, MIUnited States; ^6^VA Ann Arbor Center for Clinical Management ResearchDepartment of Family Medicine, University of MichiganAnn Arbor, MIUnited States

**Keywords:** prediabetic state, disease management, risk reduction behavior, program evaluation, patient satisfaction, attitude to computers, computers, Internet

## Abstract

**Background:**

Diabetes prevention is a national goal and particularly important in the Veterans Health Administration (VHA) where 1 in 4 veterans has diabetes. There is growing evidence to support the use of Web-based diabetes prevention program (DPP) interventions, shown to be as effective and often more feasible than in-person interventions.

**Objective:**

Our primary objective was to qualitatively explore women veterans’ early experiences with a Web-based DPP intervention. Our secondary objective was to estimate weight loss, participation, and engagement to provide context for our qualitative findings.

**Methods:**

We conducted and analyzed semistructured interviews and collected data on weight change, participation, and engagement. A total of 17 women veterans with prediabetes from a Midwest VA Women’s Health Clinic were eligible to participate; 15 completed interviews.

**Results:**

Participants perceived the DPP program as an appealing way of initiating lifestyle changes and made them feel accountable in achieving their daily goals. The online program was convenient because it could be accessed at any time, and many found that it integrated well into daily life. However, some did not like the logging aspect and some found it to be too impersonal. Participants logged in a mean 76 times, posted a mean 46 group messages, and sent a mean 20.5 private messages to the health coach over 16 weeks. Participants lost 5.24% of baseline weight, and 82% (14/17) of participants completed at least 9 of 16 core modules.

**Conclusions:**

Women veterans’ early experiences with a Web-based DPP intervention were generally positive. Accountability and convenience were key enabling factors for participation and engagement. A Web-based DPP intervention appears to be a promising means of translating the DPP for women veterans with prediabetes.

## Introduction

Type 2 diabetes is associated with significant morbidity and mortality. Nationally, diabetes affects 11% of women 45-64 years of age [1], but the prevalence has been estimated at 10% for women veterans between 45-54 years of age and 18% for those 55-64 years of age [2]. For women veterans, the burden of diabetes is further compounded by known gender disparities in the control of important modifiable risk factors shown in both Veterans Affairs (VA) [3] and non-VA studies [4,5].

Several randomized controlled trials, including the Diabetes Prevention Program (DPP) study, have demonstrated that lifestyle interventions promoting weight loss and increased physical activity significantly reduce the risk of progression to diabetes compared to placebo [6]. These landmark findings have been shown to persist up to 10 years in longitudinal observational studies [6-12]. However, translation of DPP-based lifestyle interventions has presented several challenges [13-15]. The most notable challenge is the substantial investment required to deliver—and for participants, attend—16 in-person lifestyle coaching sessions [13,16,17], resulting in significant barriers to reach and uptake among both health care systems and patients.

Women have repeatedly identified competing demands (such as caregiving) as a significant barrier to lifestyle intervention adherence [18,19]. For women veterans, distance may also be a barrier because up to one-third live in rural or highly rural areas [20]. Therefore, the time and expense of traveling to attend in-person interventions is likely to constrain participation. Furthermore, studies have shown that women veterans are more reluctant to regularly attend in-person VA-sponsored programs due to the nature of the predominantly male environment [21]. They may also feel uncomfortable discussing weight and exercise in groups that include face-to-face interaction with men [22].

Web-based DPP interventions have the potential to reduce or eliminate these barriers. Web-based DPP interventions are delivered asynchronously and are easily accessed, affording women greater convenience and flexibility. Web-based interventions can improve behavioral outcomes including increased exercise time, increased knowledge of nutritional status, and 18-month weight loss maintenance [23]. In addition, Web-based DPP interventions can produce similar weight loss compared to in-person interventions, but at a lower cost [13]. Thus, Web-based DPP interventions may be a feasible means of increasing reach and uptake of DPP interventions in the VA and an especially appealing option for women veterans with prediabetes.

To date, most of the literature related to DPP interventions has been quantitatively focused with little attention paid to participant experiences [13,24-30]. Overall, our goal was to generate rapid and relevant data on the early experiences of participants to inform future work in this area. Specifically, our primary objective was to qualitatively explore advantages and disadvantages of delivering a DPP in a Web-based format to the earliest cohort of women veterans with prediabetes in our study. Our secondary objective was to quantitatively estimate weight loss, participation, and engagement in this cohort to provide context for our qualitative findings. The rising prevalence of both prediabetes and diabetes, as well as the variable reach and uptake of in-person DPP, render our findings timely and relevant inside and outside VA.

## Methods

### Setting and Design

#### Overview

**Figure 1:**
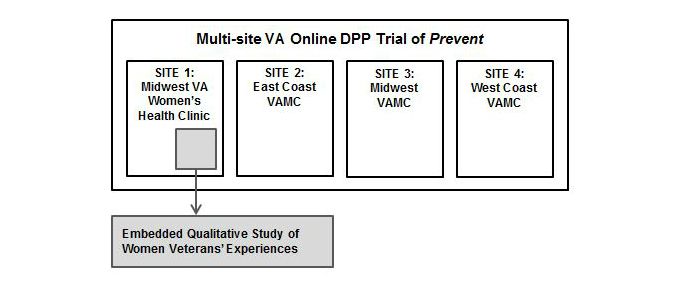
Study design overview.

This qualitative study was embedded within a larger, multisite VA trial of a Web-based DPP intervention, which used a commercially available Web-based, DPP-based group lifestyle intervention known as *Prevent*, described below. See Figure 1 for a diagram of the study design overview.

#### Multisite VA Online DPP Trial

The Multisite VA Online DPP Trial was funded by the VA Diabetes QUERI (Quality Enhancement Research Initiative) and included 4 VA Medical Centers (VAMCs). One participating VAMC—a Midwest VA Women’s Health Clinic—recruited only women veterans; the other 3 VAMCs in the study did not make a special effort to recruit women. The trial was initiated to explore alternative methods for DPP delivery. VA research funds were used to pay for 240 participant enrollment fees for 1-year access to Prevent, a commercially available Web-based diabetes prevention program developed by Omada Health (Omada Health, San Francisco, California).

**Figure 2:**
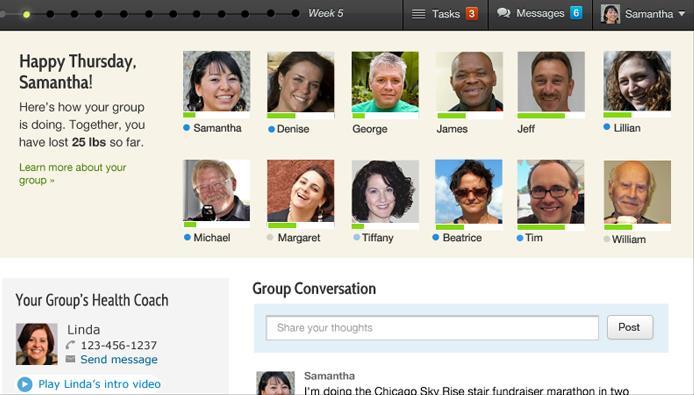
Prevent screenshot.

Prevent integrates educational modules, health coaching, and tracking tools [26]. A unique feature of this program is that it leverages social media principles to deliver a virtual DPP in a small group format, as shown in Figure 2.

Prevent has been shown to meet the Centers for Disease Control and Prevention (CDC) Diabetes Prevention and Recognition Program outcome standards [31] and weight loss outcomes of other DPP translations [26]. Prevent participants are assigned to small virtual groups based on a proprietary algorithm that uses individual characteristics including age, body mass index (BMI), and geographic location.

A 1-year membership to Prevent consists of 16 weekly modules of intensive core curriculum followed by an 8-month maintenance phase (8 “post-core” monthly modules). Over the first 16 weeks, a new core module is made available each week with new goals and encouragements to post responses to interactive exercises. Participants can choose when to log in and for how long. A certified professional health coach, affiliated with the Prevent program, is assigned to each group to help deliver the curriculum, answer questions, and monitor group interactions on a regular basis to ensure an appropriate and positive virtual group environment. Participants can post messages to the entire group or send private messages to the health coach. All participants receive a pedometer, food and exercise trackers, and a wireless scale that automatically uploads weight data to Prevent on a daily basis.

Omada Health agreed to transfer all participation data from consented study participants to the VA research team for analysis. The study team analyzed all data independently and retained sole authority over all publication-related decisions throughout the course of the study. Enrollment for the multisite VA DPP trial began in October 2013.

#### Embedded Qualitative Study of Women Veterans’ Experiences

We used an exploratory mixed-methods study design to examine the early experiences of a subset of women veterans with prediabetes enrolled in the multisite VA online DPP trial [32].

### Participants and Recruitment

**Figure 3:**
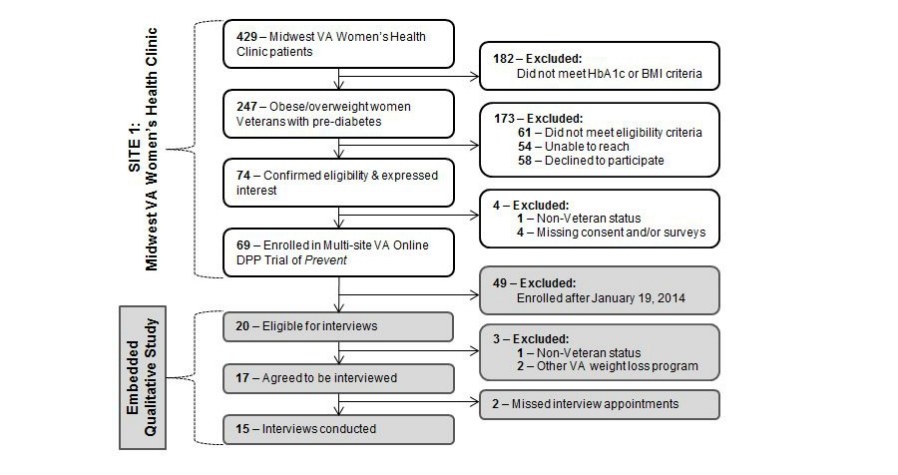
Study recruitment details.

Women veterans with prediabetes from the Midwest VA Women’s Health Clinic who had enrolled in *Prevent* by January 19, 2014, were invited to participate in interviews for this embedded qualitative study (Figure 3). Prediabetes was defined as fasting plasma glucose of 100-125 mg/dl or a hemoglobin A1c (A1c) of 5.7-6.4% in the past 12 months. All participants were obese (BMI >30 kg/m^2^) or overweight (BMI >25 kg/m^2^) with one cardiovascular risk factor (eg, hypertension). Participants with diabetes or eating disorders, use of antiglycemic medications (including metformin), pregnancy or a plan to become pregnant, a medical contraindication to lifestyle modification, lack of regular access to a computer with an Internet connection or email address, or participation in a VA weight management program within the prior 6 months were excluded.

Eligible participants were mailed invitation letters from their primary care providers and received follow-up phone calls from research staff. All participants were asked to sign informed consent. Consented participants were then assigned to a Prevent group on a rolling basis; groups included veterans and non-veterans and female and male participants.

Beginning in February 2014, the research team contacted women veterans with prediabetes from one Midwest VA Women’s Health Clinic who had enrolled in Prevent by January 19, 2014, for interviews. All participants who expressed interest were scheduled for interviews with the investigator (JS). All participants who completed an interview received a US $25 gift card.

### Data Collection and Analysis

Qualitative data were collected through in-person semistructured interviews conducted in a private room by one investigator (JS) in March 2014. A copy of the interview guide is provided in Multimedia Appendix 1. We audio-recorded the interviews, which lasted 30-50 minutes, transcribed them verbatim, and manually coded the transcripts using a content analysis approach [33]. Codes were developed inductively by coding the first 6 interviews using a consensual process. Descriptive, inductive content analysis was used to identify common themes. Two coders independently coded each interview manually (JS, KE, KH, EV, TM) and analyzed them for common themes. While it was not a criterion for stopping interviews, we did reach thematic saturation. The analysis team (KE, KH, EV, TM) met regularly to iteratively reach consensus on any coding discrepancies [34].

General health information was collected from medical records (age, A1c, BMI, weight, and service connection) and an enrollment questionnaire (race/ethnicity, income, education, employment status, comorbidities, and self-rated health status). Weight data were objectively collected using data uploads from wireless scales. We assessed participation based on frequency of logins to Prevent, weight assessments, and messages sent. Engagement was assessed based on rates of module completion and mean percent weight loss over 16 weeks**.**

The study was approved by the local and coordinating center’s Institutional Review Boards, and all participants completed a written informed consent process.

## Results

By January 19, 2014, 20 women veterans with prediabetes had enrolled in Prevent. Our sample included the first 17 interview-eligible participants who enrolled in the program. All 17 agreed to be interviewed but two did not show up for interviews, leaving a final qualitative sample of 15 participants.

At baseline, participants had a mean age of 56.8 years, BMI of 35.6 (5.3) kg/m^2^, and A1c of 6.0% (0.2) (Table 1); 41% were African American (n=7).

**Table 1:** Participant baseline^a^ characteristics (n=17).

Characteristics		n (%) or mean (SD)
Age (years), mean (SD)		56.8 (7.0)
**Race or ethnicity** ^b^ **, n (%)**		
	White	8 (47)
	Black	7 (41)
	Did not disclose	2 (12)
**Education, n (%)**		
	Some college or 2-year degree	11 (64)
	4-year college graduate	2 (12)
	˃4-year college degree	3 (18)
	Did not disclose	1 (6)
**Annual income, US $** ^b^ **, n (%)**		
	<50,000	8 (47)
	≥50,000	6 (35)
	Did not disclose	3 (18)
**Employment status, n (%)**		
	Working part-time/full-time	7 (41)
	Unemployed/disabled/unable to work	3 (18)
	Retired	6 (35)
	Did not disclose	1 (6)
**Comorbidities**		
	Number^c^, mean (SD)	1.9 (0.7)
	Hypertension, n (%)	8 (47)
	Mental health conditions^d^, n (%)	5 (30)
**Self-rated general health status, n (%)**		
	Good or better health	14 (82)
	Fair or poor health	3 (18)
**Physiological tests, mean (SD)**		
	A1c	6.0 (0.2)
	BMI	35.6 (5.3)
	Weight (lbs)	210.4 (38.6)

^a^Baseline characteristics are from surveys and labs taken prior to the first Web-based DPP module.

^b^One or more missing values.

^c^Comorbidities included hypertension, hyperlipidemia, coronary heart disease, heart failure, liver disease, lung disease, stroke, arthritis, and/or osteoporosis.

^d^Mental health conditions included depression, posttraumatic stress disorder, schizophrenia, and/or bipolar disorder.

### Qualitative Findings

#### Overview

Qualitative interview data provided insights into the early experiences of women veterans in the Web-based DPP intervention. The women were enthusiastic about Prevent and described their experiences, highlighting both facilitators and challenges. We identified seven broad themes that emerged from the 15 interviews.

#### Theme 1: The Program Is a Good Fit With Perceived Health Needs

Overall, participants perceived the Web-based DPP as a good fit for their health needs. One participant stated: “I was just thinking and praying about the fact that I need to get my weight under control. Gotta get my health under control and I was just feeling so yucky and this program came along. And it was just perfect” [ID11]. Another participant explained: “You know, I’ve battled my weight all my life, you know, and I knew it was time to get back and start doing something but just kind of casting around and trying to find the right program and the right fit. So this [program] sounded good” [ID13].

#### Theme 2: The Program Is Convenient

Almost all participants emphasized the convenience of a Web-based delivery format. One participant explained:

Knowing I didn’t have to leave out the house (sic); knowing I can participate with other people just via Internet, this was great. It was great doing [the program] by Internet. You didn’t have to get up and go places all the time and do things and [could] keep…your own pace. The only thing I had to get up every morning and do is weigh in. That’s not a problem so far.

ID6

Another participant highlighted the flexibility of the Web-based format: “…‘unstructuredness:’ Ability to go on at midnight and see what’s going on. Not [being] tied to a schedule” [ID5].

#### Theme 3: The Program Integrates With Daily Life

Participants stressed the ease with which they could integrate the intervention into their existing daily routines and how integration in daily activities increased participation and engagement:

I get on the scale every day. That’s a no-brainer ‘cause it’s in the bathroom, you know, after I brush my teeth and all that stuff just before I get in the shower I get on and then I get in the shower and I’m going about my day. No brain. Easiest thing in the world to do. So I’ve never missed getting on the scale.

ID8

Several participants specifically commented on the increased convenience of the Web-based format compared to previously attempted in-person programs, such as “I don’t have to drive into the [medical center] every, you know, every day, that kind of thing. I have had to do that with other programs in the past and it’s just, it can take up a lot of my time in the day” [ID7].

#### Theme 4: “I Feel Accountable”

All participants felt accountable to the Web-based DPP intervention, which was a significant motivation to meet daily goals including daily weighing. One participant stated:

I think that the program helped a lot. When I made a commitment to weigh myself every day that was huge, you know, that kept me honest. On the days I really didn’t want to get on the scale—which was the day I really needed to be on the scale—that was good. Knowing I was sort of accountable to the program, that was a big motivator.

ID13

Another explained: “It’s the accountability. A lot of this program is because of the accountability, having to put everything, having to get on the scale every morning, and I sleep and walk with my pedometer and to get my 5000 steps or more in a day and things like that” [ID11].

One participant stated: “Um…the daily weigh-in I think for me was just, was the biggest. Really the biggest and knowing that I was a part of a group and that we were all working towards the goals so I was part of something, was a big motivator. Having that goal set was also helpful” [ID13]. Another participant explained:

Well, there’s a little bar that says what the group’s goal is for steps and then there’s like a, I think it’s a green bar, that shows everybody’s steps, and then there’s like a little tiny bar that’s you. So it will tell you how many steps if you hover over it. So I’ll take and figure out the percentage because…I always want to look to make sure I’m keeping up my steps so I’m not the slacker in the group.

ID10

#### Theme 5: “I Hate Logging”

Though tracking and entering data fosters accountability, it also proved to be a deterrent for some participants. One example included the food and exercise trackers, which are ubiquitous in most weight loss programs whether they are online or not. Some participants did not welcome the logging that was needed, as described by one participant: “I’m not one to log. I hate logging stuff. I can’t stand to log. I’m lazy. When it comes up to logging my activities, you know. I know what I’m doing, but I don’t want to log my life. I’m not interested in logging my life” [ID4].

#### Theme 6: “If the Program Were In-Person, My Group Would Know Me Head-to-Toe”

Last, several participants viewed the Web-based group format as less interactive or intimate, as highlighted by one participant:

I’m a real talker. So if we were sitting in a room face-to-face, they’d know me from head to toe by now. Sitting before people I seem to be a little bit more open than online. Probably simply because I’m very conscious of my wording and so if I sit before you I may say a couple paragraphs, but typing it’ll probably be a couple of sentences.

ID8

Another participant said: “Online: I didn’t care for it. If we were all in one room it would be a different situation, you know. Body language says a lot to me. I mean you can say all the right words you want in black and white but body language tells me a lot” [ID14].

#### Theme 7: Difficult to Figure It Out

Computer literacy made the Web-based format more challenging for some, including one participant who stated: “There were some parts of the website I guess that I never did figure out. Well, I’m sure it’s user friendly for most people. Not all of us have the same abilities when it comes to that” [ID5].

Several participants also described technical and/or equipment failures that interfered with engagement. “For instance, my scale got goofed up and I didn’t know how to fix it and she [the health coach] finally helped me figure out what to do and it worked”. Despite several participants encountering technical issues, most of them were resolved through responsive support services and generally, the convenience of the Web-based delivery format outweighed technical challenges. “My computer acts up a lot, so I don’t get to log in and do all the stuff that they would like me to do, but versus going to meetings and all of that, I would prefer to do it online” [ID9].

At 16 weeks, participants lost an average of 5.24% (SD 0.05) from starting body weight. All participants completed at least 4 core modules, and 82% (14/17) completed at least 9 of the 16 core modules. On average, participants logged into the Web-based intervention 76 (SD 64.3) times, weighed in 89 (SD 34.8) times, posted 46.5 (SD 35.7) group messages, and sent 20.5 (SD 9.9) private messages to the health coach over 16 weeks (Table 2).

**Table 2:** Participation, engagement, and weight change over 16 weeks (n=17).

Measure		Result
**Participation metrics over 16 weeks per participant, mean (SD)**		
	Logins^a^	76 (64.3)
	Weight assessments^b^	88.5 (34.8)
	Comments left on discussion board	46.5 (35.7)
	Private message to health coach	20.5 (9.9)
**Completion of 16 weekly core modules, n (%)**		
	Participants who completed at least 9 of 16 of modules	14 (82)
	Participants who completed all 16 modules	7 (41)
**Percent weight change from starting weight over 16 weeks, %**		
	All participants (n=17)	5.24
	For participants completing ≥9 modules (n=14)	5.92
	For participants completing all 16 modules (n=7)	8.59

^a^Logins refers to the number of times participants logged in to the program.

^b^Weight assessments were conducted using a home wireless scale.

## Discussion

### Principal Results

Our qualitative results showed participants perceived the Web-based DPP as an appealing way of initiating lifestyle changes. The program was convenient because it could be accessed at any time, it integrated well into daily life, and it made them feel accountable in achieving their daily goals. One way the program helped make participants feel accountable was through regular and ad hoc interactions with the health coach and members of their group. Participants posted messages about their progress (or lack thereof), which elicited supportive feedback from other group members and/or the health coach. Group members could see the progress each participant was making toward her goals (Figure 2). In addition, group progress was updated daily and helped participants see their contribution to their group’s progress as a whole. However, some did not like logging and some felt the program was too impersonal. Our quantitative results indicated high levels of participation and engagement as measured by rates of educational module completion and the frequency of online interactions. These high levels of participation and engagement, including self-monitoring, are likely to have contributed toward weight loss [35-37].

Generally, little is known about participant experiences in diabetes prevention programs, which is an important gap in the existing literature [13,25-30], and even less is known about Web-based DPP interventions. Based on early experiences of a small cohort of women veterans with prediabetes, our findings suggest that a Web-based DPP intervention may be an especially appealing option for them. The challenges of delivering an in-person DPP intervention coupled with the rising prevalence of prediabetes and diabetes, place increasing urgency on learning about viable options to engage patients in effective non-traditional programs.

Our results provide several insights for future practice. First, offering a Web-based version of DPP may be an effective way to increase the repertoire of diabetes risk reduction strategies. From a health system perspective, a Web-based DPP intervention may be more feasible and sustainable to implement on a large scale given lower operating costs. The VA is a regionalized health care system where Internet-based health care delivery has great potential to increase access and sustainability of programs targeted to preventing diabetes. However, rates of health-related Internet use are relatively low among veterans [38,39]. Higher education and urban location are strongly and positively associated with veterans’ health-related Internet use; even after controlling for socioeconomic characteristics, interventions may be needed to increase use among less educated and rural veterans [40].

Despite these challenges, our sample, albeit small, provides early evidence that well-designed Web-based DPP interventions may be welcomed and effectively utilized by women veterans. In fact, a Web-based delivery format may help increase participation and engagement in a DPP or weight management programs for all patients; traditional onsite programs struggle with low levels of participation in real-world settings [41-43]. A Web-based DPP intervention may increase enrollment, participation, and satisfaction among patients who are not able to participate in onsite programs because of distance, work schedules, or discomfort.

Second, unlike in-person DPP interventions with established standards for delivery, Web-based interventions are relatively new and necessitate evaluation. It is not yet clear how best to optimize this type of DPP delivery to enhance desired clinical outcomes. Evidence from our qualitative data suggests that accountability and automated real-time monitoring affected participants’ early experiences and encouraged participation and engagement. Accountability was frequently described as a sense of obligation to the group or the health coach. Technological features of Prevent helped reinforce this feeling of accountability. A wireless scale automatically uploaded weight data and online tools displayed visual progress toward goals, individually and aggregated for the group in real-time. In contrast, diet and exercise trackers that required manual data entry were inconsistently used or not used at all. Thus, our early findings suggest that Web-based DPP interventions should strive to include elements of accountability and automated monitoring systems whenever possible.

Our qualitative results also allude to an important shortcoming that may occur in a virtual online group environment. The Web-based format felt less intimate to some participants who, for example, highlighted the importance of non-verbal communication that is possible only with face-to-face interventions. This raises interesting questions about the potential merging of in-person and Web-based programs to best meet patient needs while also maximizing available resources. For example, individuals assigned to a Web-based group might benefit, if geographically feasible, from having the option of a limited number of face-to-face meetings with their group. Future studies should help establish minimum standards and best practices for Web-based DPP delivery to help answer these critical questions.

### Limitations

Our findings should be interpreted with several limitations in mind. First, our sample size was small. However, our qualitative data indicated thematic saturation (ie, no new themes emerged in the last five interviews that were analyzed). The small sample size limits generalizability of quantitative results, including estimates of weight loss, and will need to be confirmed in a larger study; these were included here to provide context for the qualitative results. Second, enrollment occurred on a rolling basis and our sample included the first 17 interview-eligible participants who enrolled in the program. These first participants may have been more highly motivated patients who completed the multistep recruitment process relatively quickly. It would be important to continue to assess participant experiences and collect data on reasons for non-participation in future studies.

Our participants were enrolled in the Web-based DPP intervention for a relatively short timeframe, so further studies are needed to assess longer-term experiences and outcomes. Our findings are also less generalizable because we included only women veterans from one VA Women’s Health Clinic. Further studies are needed to assess the extent of applicability of these findings to other settings and populations. Last, comparing and contrasting qualitative and quantitative findings was outside the scope of this small study but will be the focus of future work in this area.

### Conclusions

In conclusion, a Web-based DPP intervention appears to be a promising means of translating the DPP for women veterans with prediabetes in the VA. Our early qualitative findings provide a deeper understanding of participants’ early experiences and reveal how the convenience, fit, and integration of the program into daily life, and feelings of accountability contributed to participation and engagement. Our quantitative findings demonstrate high levels of participation, engagement, and meaningful weight loss, which is often a challenge with in-person interventions. These findings are particularly valuable given the paucity of literature in this domain and the high prevalence of prediabetes [44]. Studies with larger and more diverse cohorts/settings, non-completers, and long-term follow-up are needed to provide a more definitive evidence base for Web-based DPP interventions.

## Multimedia Appendix 1

Patient interview guide.

## Abbreviations

A1c: hemoglobin A1c
BMI: body mass index
CDC: Centers for Disease Control and Prevention
DPP: Diabetes Prevention Program
QUERI: Quality Enhancement Research Initiative
VA: Veterans Affairs
VAMCs: VA Medical Centers
