# Supplementary material for: Women Veterans’ Experience With a Web-Based Diabetes Prevention Program: A Qualitative Study to Inform Future Practice
Source: J Med Internet Res. 2015 May 25;17(5):e127. doi: 10.2196/jmir.4332 (PMC4468391; doi:10.2196/jmir.4332)
Supplement: Supplementary file 1 [file jmir_v17i5e127_app1.pdf]

Multimedia Appendix 1. Online Diabetes Prevention Program: Patient Interview Guide

Construct	Question	Probes
Enrollment: I would like to start with a question about your reasons for enrolling in Prevent.		
Enrollment	What made you decide to participate in Prevent?	• What appealed to you about the program?<br>• Did you think you needed to make any lifestyle changes?<br>• How did the diagnosis of pre-diabetes impact your decision to enroll?<br>• How did the fact that the program was online influence your decision?
Relationships: Now I want to talk about your relationships with your health coach and the members of your group.		
Relationships	What did you think about having a health coach?	• How would you describe your interactions with the health coach? Can you give me an example of a positive interaction? A negative interaction?<br>• Did you ever talk with or correspond with your health coach one-on-one about something you weren't comfortable mentioning in the online forum?<br>• Was your health coach responsive to you?
	What did you think about being in a small group?	• How would you describe your interactions with the group? Can you give me an example of a positive interaction? A negative interaction?
	What relationships did you develop?	• Do you think you will stay in touch with these people?
	How did these relationships impact your experience with Prevent?	• In what ways were these relationships supportive? Not supportive?
	How were these relationships affected by the group being conducted online?	• Was there anything about the online experience that made things more or less comfortable than a face-to-face interaction?
General Assessment, Goals, and Level of Satisfaction: Now I want to find out how Prevent is going in general.		
General Assessment	Can you tell me how things have been going for you so far in Prevent?	• Has the program made you more aware of your eating patterns? Can you give me some examples? Are you keeping a food diary?<br>• Has the program made you more aware of your physical activity? Can you give me some examples? Are you keeping a log of your activity?

Construct	Question	Probes
Goals	What goals did you set for yourself?	• Do you remember the goal to reduce your body weight by 7% and complete 150 minutes of physical activity each week?<br>• If patient did not set goals: Why didn't you set goals? Did you take another approach?
	Have you reached your goals to a satisfactory level?	• What hindered you from reaching your goals?<br>• What helped you reach your goals?
Satisfaction	What was the biggest change you made because of your participation in Prevent?<br>What did you like about Prevent?<br>What did you dislike about Prevent?	
	What would you change about Prevent?	• If this change had been implemented, how would this have affected you?
Sustainability: Now I want to ask you some questions about being able to sustain the changes you have made in the program.		
Sustainability	Do you think the changes you've made will be something you'll be able to continue to do on your own?	
Closing: That is all of my questions. Is there anything you would like to add? If no, I'd like to thank you for taking the time to share your experiences with us. Remember to continue logging in to Prevent and participating until your 1-year membership expires.		
